# Eg5 and Diseases: From the Well-Known Role in Cancer to the Less-Known Activity in Noncancerous Pathological Conditions

**DOI:** 10.1155/2024/3649912

**Published:** 2024-06-20

**Authors:** Alessia Ricci, Simone Carradori, Amelia Cataldi, Susi Zara

**Affiliations:** Department of Pharmacy, University “G. d'Annunzio” Chieti-Pescara, Chieti, 66100, Italy

## Abstract

Eg5 is a protein encoded by KIF11 gene and is primarily involved in correct mitotic cell division. It is also involved in nonmitotic processes such as polypeptide synthesis, protein transport, and angiogenesis. The scientific literature sheds light on the ubiquitous functions of KIF11 and its involvement in the onset and progression of different pathologies. This review focuses attention on two main points: (1) the correlation between Eg5 and cancer and (2) the involvement of Eg5 in noncancerous conditions. Regarding the first point, several tumors revealed an overexpression of this kinesin, thus pushing to look for new Eg5 inhibitors for clinical practice. In addition, the evaluation of Eg5 expression represents a crucial step, as its overexpression could predict a poor prognosis for cancer patients. Referring to the second point, in specific pathological conditions, the reduced activity of Eg5 can be one of the causes of pathological onset. This is the case of Alzheimer's disease (AD), in which A*β* and Tau work as Eg5 inhibitors, or in acquired immune deficiency syndrome (AIDS), in which Tat-mediated Eg5 determines the loss of CD^4+^ T-lymphocytes. Reduced Eg5 activity, due to mutations of KIF11 gene, is also responsible for pathological conditions such as microcephaly with or without chorioretinopathy, lymphedema, or intellectual disability (MCLRI) and familial exudative vitreous retinopathy (FEVR). In conclusion, this review highlights the double impact that overexpression or loss of function of Eg5 could have in the onset and progression of different pathological situations. This emphasizes, on one hand, a possible role of Eg5 as a potential biomarker and new target in cancer and, on the other hand, the promotion of Eg5 expression/activity as a new therapeutic strategy in different noncancerous diseases.

## 1. Introduction

**Figure 1:**
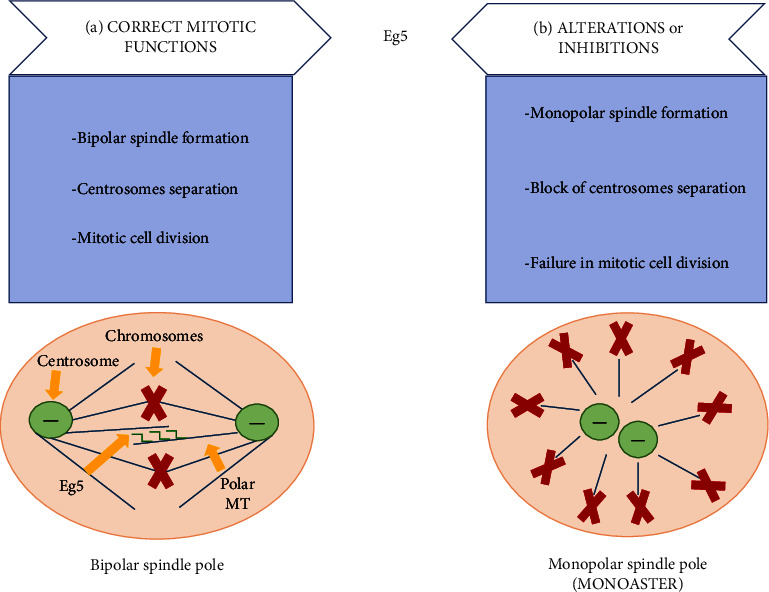
Eg5 activity in mitosis. (a) The correct activity of Eg5 (dark green) allows the overlapping of two polar microtubules at the equator of the cell and the separation of centrosomes at the opposite poles of the cell, thus obtaining a bipolar spindle pole formation. (b) Eg5 alterations or inhibitions determine monopolar spindle formation (monoaster).

Kinesin Eg5, also known as Kinesin Spindle Protein (KSP) or KIF11 (from the gene that encodes Eg5), is a motor protein that belongs to the Kinesins superfamily (KIFs) 11. It is a motor protein associated with microtubules (MTs), as it uses the structure of MTs as a road and the hydrolysis of ATP into its motor domain as a source of energy to move along the microtubules [1]. Eg5 is primarily identified as a mitotic motor protein; in fact, its essential activity in the formation of bipolar spindles during mitotic cell division has been widely recognized. Between the prophase and prometaphase, the mitotic spindle MTs, starting from the two centrosomes, extend across the cell cytoplasm. Eg5 reaches maximum concentration during these steps; thanks to its homotetrameric structure, anchors itself to the plus end extremity of interpolar MTs, allowing its overlapping at the equator of the cell and, at the same time, the separation of the two centrosomes at the opposite poles of the cell [2, 3]. To allow bipolar spindle formation, different mechanisms of action coexist, such as phosphorylation or deacetylation of different Eg5 sites [4]. Before entering mitosis, Eg5 is acetylated on lysine 890 in the tail domain and this form of Eg5 is inactive; to become active, a deacetylation is made by Histone Deacetylase 1 and a mitotic progression is possible [5]. Then, two additional phosphorylations on the tail domain of Eg5 should be required: the first on the Thr-927, made by cyclin-dependent kinase 1 in prophase, allows the localization of Eg5 to centrosomal MT, where it accumulates. Then, phosphorylation made by Nek6/Nek7, two serine-threonine protein kinases, can initiate centrosome separation and ultimately spindle bipolarity [6]. Simultaneously, motor activity is regulated through phosphorylation of three tyrosine residues within the motor domain (Y211, Y125, and Y231) produced by kinases of the Src family (SFK) [7]. All these mechanistic processes determine bipolar spindle pole formation, followed by correct mitotic cell division (Figure 1(a)). Perturbations in Eg5 functions or perturbations in the phosphorylation process of Eg5 activation determine the formation of a monopolar spindle, called a monoaster, with failure in mitotic cell division (Figure 1(b)) [3]. This failure in the mitotic process and the formation of monopolar spindle, inevitably, trigger a programmed cell death. The condition by which the cells with altered Eg5 functions are unable to conclude the mitotic event is called “mitotic catastrophe” and triggers the apoptotic cascade as preferential programmed cell death [8, 9].

**Figure 2:**
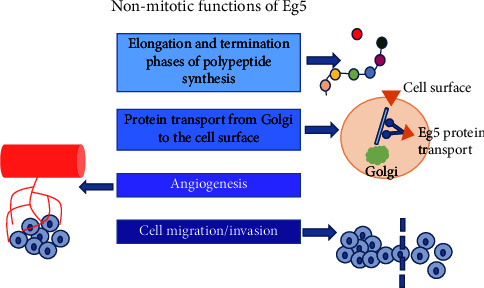
Nonmitotic activity of Eg5. Schematic summary of the main nonmitotic Eg5 functions reported in the literature.

In addition to the mitotic function, during the last decade, nonmitotic functions, correlated with an interphase pool of Eg5 in the cells, were highlighted (Figure 2).

In 2011, Bartoli et al. discovered that Eg5 is required for physiological levels of protein synthesis. It is likely that this kinesin is connected to ribosomes during the interphase and its activity is essential during the postinitiation phase of polypeptide synthesis (elongation/termination) by linking 80S ribosomes subunits to MTs during translation. More specifically, Eg5 serves as a motile link between the ribosome and MTs and its *in vitro* inhibition is known to determine errors in the elongation and termination steps. How Eg5 binds to the ribosome is still unclear: a direct binding of Eg5 to the ribosome (Eg5 as a direct link between 80S of the ribosome and MTs) or an indirect binding (through an undefined linker molecule) are hypothesized [10]. In addition to the role in polypeptide synthesis, another nonmitotic function identified for Eg5 is in protein transport from the trans-Golgi complex to the cell surface: Eg5 interacts with the protein carrier, namely CARTS, and moves proteins, through CARTS, from the Golgi to the cell surface. Eg5 pharmacological inhibition determined an interruption of the activity of the CARTS carrier [11]. Eg5 is also extensively studied as a critical protein that regulates angiogenesis, axonal branching, and cell motility, particularly in cancer models [12–14]. However, it is unclear how Eg5 regulates these events. With respect to the relationship between Eg5 and angiogenesis, it has been elucidated that Eg5 is necessary for normal and abnormal vascular development. An upregulation of Eg5 was found in blood endothelial cells and lymphoblasts, as after angiogenic stimulation of chick embryos with VEGF-A, and also in tumor blood vessels. *In vitro* pharmacological inhibition of Eg5 decreases endothelial cell proliferation and migration, while in *in vivo* zebrafish and chick embryos models, interference with Eg5 function causes developmental and vascular defects, along to inhibition of angiogenesis in tumor models [13]. At the same time, Eg5 influences the migration of different types of cells, such as neurons and tumor cells. In neurons, Falnikar et al. showed an Eg5 regulatory potential for the migration process and the interruption of migration when the neuron reaches its destination [15]. In tumor cells, such as human pancreatic cells, pharmacological inhibition of Eg5 decreases cell migration and invasion [12].

The highest number of published articles on Eg5 is related to the role of this kinesin in cancer. High Eg5 expression levels have been highlighted for decades in different tumors, with a poor prognosis for patients and pharmacological Eg5 inhibitors were tested as a new therapeutic strategy. However, this protein plays an important role in other less well-known diseases, for which a stimulation of Eg5 expression could be a valid therapeutic approach, even if, compared to the high amount of tested Eg5 inhibitors for cancer treatment, Eg5 activators are currently not available.

**Figure 3:**
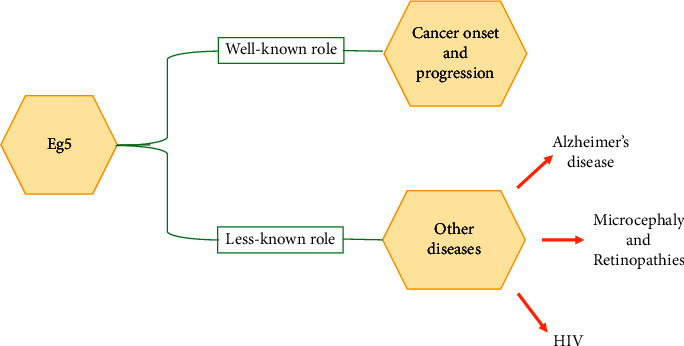
Schematic representation of the aim of this review: summarizing recent findings on the well-known activity of Eg5 in cancer and recent and old findings on the less well-known role of Eg5 in other diseases.

The aim of the present review (Figure 3) is, on the one hand, to summarize recent findings on the correlation between Eg5 and the onset and progression of cancerous malignancies, and, on the other hand, to shed light and summarize old and recent findings on the less known correlation between Eg5 and specific diseases, such as Alzheimer's disease (AD), the genetic form of microcephaly typically associated with retinopathy, genetic retinopathy conditions, and others.

## 2. Eg5 in Tumor Onset and Progression

The correlation between Eg5 expression and cancer origin and progression has been well known for decades and is related to an unfavorable prognosis [16–18]. Indeed, Eg5 expression levels in nontumor proliferative tissues are significantly lower than those recorded in tumor tissues, allowing to consider this protein as a possible prognostic biomarker and a therapeutic target. Nowadays, the scientific community is still focused on searching for Eg5 inhibitors able to counteract tumor onset and progression. Several recent reviews focused attention on the role of Eg5 inhibitors as a cancer therapeutic strategy, highlighting new findings on this topic [19–23]. As all recent reviews on Eg5 and cancer are mainly focused on the inhibition of this protein and the discovery of new drugs, in this section, the current literature, from 2021 to 2023, is discussed on the correlation between Eg5 and different cancerous malignancies (resumed in Table 1), from a biological point of view.

**Figure 4:**
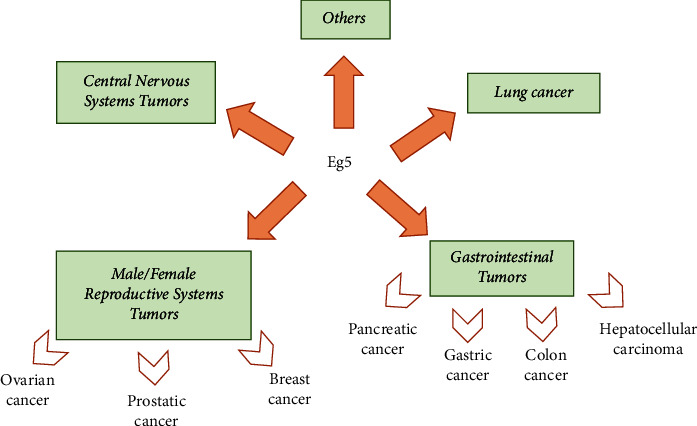
Schematic summary of the main cancerous conditions in which high expression of Eg5 was found and reported in the literature between 2021 and 2023.

The literature of the last 2 years shed light on lung and gastro-intestinal tract carcinomas as tumors with the highest correlation with Eg5 expression, followed by reproductive system carcinomas (breast, ovarian, and prostate) and central nervous system tumors; in other tumors (such as bladder cancer, renal cancer, and thyroid cancer) Eg5 overexpression is known, but less papers are available. Based on the abundance of literature information regarding Eg5 and these tumors, we started our discussion with those tumors for which more information is available, followed by tumors with a less known correlation with Eg5 (Figure 4).

### 2.1. Lung Carcinoma

**Figure 5:**
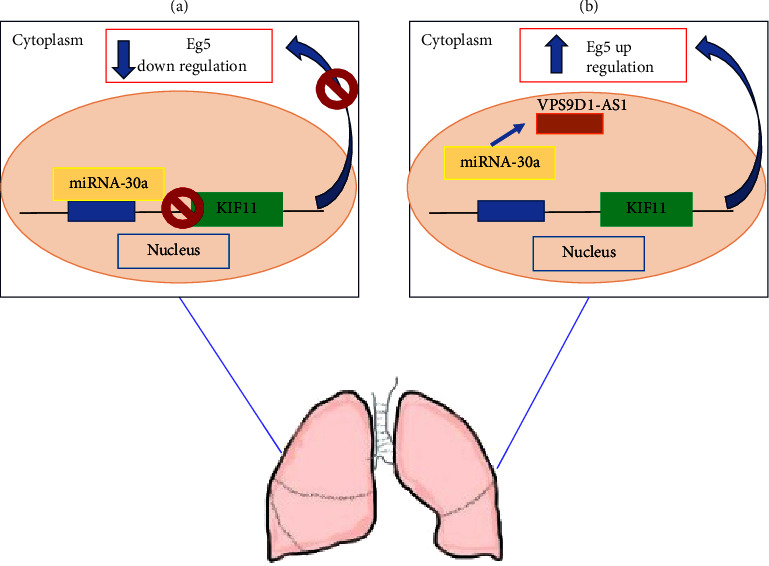
(a) In normal conditions, miRNA-30a counteracts tumor onset and progression by inhibiting Eg5 synthesis. (b) In tumors, such as LUAD, the lnc-RNA VSP9D1-AS1 sponges miRNA-30a activity induces gene expression and protein synthesis of Eg5. Blue arrows in (a) and (b) represent Eg5 release within the cytoplasm.

Lung cancer is one of the most common tumors worldwide. In 2020, it was the second, after female breast cancer, for the number of new cases (11.7%) and, despite the available therapeutic approaches, it remained the leading cause of cancer mortality (18%) [67]. Non-small-cell lung carcinoma (NSCLC) is the most widespread and common type of lung cancer and represents more than 80% of the total number of lung cancers, with lung adenocarcinoma (LUAD) and lung squamous carcinoma (LUSC) being the most common subclasses [68]. The necessity of new targets to hit is undoubtedly indispensable to improve the life expectancy of people. A consistent number of findings between 2021 and 2023 have identified Eg5 as a prognostic factor and a possible new therapeutic target for patients affected by lung cancer. An integrated bioinformatic data set analysis of 497 LUAD tissues and 54 normal tissues from the Cancer Genome Atlas (TCGA) highlighted KIF11 as a pivotal gene from the ten genes identified with the highest expression levels. KIF11 was highly expressed in LUAD compared to normal tissues and its high expression is correlated with poor overall survival and worse progression-free survival, in addition to the positive association with tumor grade. Furthermore, KIF11 expression appears to be related to the formation of immune infiltrating cells in the tumor microenvironment: Resting NK cells and memory CD^4+^ T cells, regulatory T cells, and monocytes were significantly associated with KIF11 expression and with overall survival of patients with LUAD. KIF11 knockdown determined an inhibition of proliferation, migration, and invasion of lung adenocarcinoma cells A549 and PC-9, in addition to arrest of the G2/M phase cell cycle and induction of apoptosis [24]. How KIF11 modulates the onset and progression of LUAD is not yet known. Recent interesting information is the correlation found between noncoding RNAs (nc-RNAs) and KIF11. nc-RNAs are small RNAs with no ability in protein coding, based on their length, shape, and location, and are classified in three main nc-RNAs: circ-RNA, miRNA, and long noncoding RNAs (lnc-RNA). lnc-RNAs and circ-RNAs can control gene expression by modulating transcription factors, regulating protein-protein interactions, and epigenetic modulation of chromatin; all these events play roles in tumor-related processes, such as proliferation, invasion, and metastasis [69]. What was discovered in recent years is that the progression of LUAD could be triggered and improved via the VPS9D1-AS1/miRNA-30a-5p/KIF11 axis. VPS9D1-AS1 is an lnc-RNA that, through interactions with specific miRNA, in particular by sponging different miRNA, promotes malignant tumor progression [70, 71]. In LUAD, VPS9D1-AS1 results overexpressed and appear to facilitate proliferation, migration, and invasion of LUAD cells. In this malignancy, VPS9D1-AS1 sponges a specific miRNA, miRNA-30a-5p, an endogenous noncoding RNA that normally suppresses the progression of different tumor types [72–74] and whose expression is downregulated in LUAD. KIF11 is a downstream gene of miRNA-30a-5p; the malignant phenotype of LUAD is inhibited by the ability of miRNA-30a-5p to target and inhibit KIF11 overexpression. The possibility that VPS9D1-AS1 targets KIF11, through sponge activity in miRNA-30a-5p, promoting malignant progression of LUAD, could be a new possible axis underling LUAD onset and progression (Figure 5) [26, 27].

At the same time, Balasundaram et al. focused their attention on the regulatory axis of circRNA-miRNA-mRNA in NSCLC. They identified 12 dysregulated pivotal genes associated with NSCLC, including KIF11. In particular, they analyzed the correlation between circ-RNA-miRNA-mRNA with the identified hub genes. These 12 genes encode 12 mRNAs which, in turn, are linked to 8 miRNAs associated with 1 circRNA. Regarding KIF11, they found that a specific circ-RNA (circ_0003812) is associated with miRNA-200b, which in turn is related to KIF11. This axis could influence the biological processes of NSCLC and could represent a new blockable biomarker axis [30]. Through the Kaplan–Meier survival analysis, KIF11 was also identified among the 9 hub genes related to poor overall survival in patients with NSCLC, again with high expression in tumor tissues compared to nontumor tissues [25]. A microarray data set from the Gene Expression Omnibus (GEO) database identified other 9 genes correlated with the progression of NSCLC: in the list KIF11 appears as an upregulated gene involved in tumorigenesis, progression, and resistance to cisplatin, its knockdown decreased the proliferation of A549 and SPCA1 cells [28]. KIF11 was also identified as a key gene involved in the tumorigenesis of benzo(a)pyrenediol-induced NSCLC [29].

Based on these findings reported in the last 2 years, it is possible to include Eg5 between potential biomarker for lung cancer. In LUAD diagnosis, different biomarkers are currently available: KRAS is the most frequent mutated gene with around 30% of mutation frequency, followed by EGFR (around 10% of frequency in mutation), MET (8% of mutation frequency) and ALK (4–7% of rearrangement frequency). Other biomarkers show mutations or rearrangements frequency ranging from 1% to 8%, such as ROS1, NTRK1, HER2, BRAF, DDR2, and PD-L1. In NSCLC, less biomarkers are available: FGFR shows 20% of amplification frequency, followed by 16% of PTEN gene deletion and 7% of PIK3CA gene mutation, while KRAS, EGFR, and MET show approximately 5% of mutations frequency [75]. To obtain this panel of biomarkers could represent a crucial step to fine tune a tailored target therapy. For instance, patients with LUAD KRAS G12C mutated are treated with KRAS inhibitors, while patients with PD-L1/PD1 are eligible for immunotherapy. However, for most of mentioned biomarkers, the mutation percentage is very low and, resistance processes are often responsible for low response to therapy. In addition, for NSCLC, less biomarkers are available compared to LUAD. Eg5 could become a new valid target to enlarge the panel of available biomarkers. Furthermore, focusing on this point, Eg5 expression can be deeply investigated between LUAD and NSCLC thus allowing to distinguish between the two tumor types.

### 2.2. Gastrointestinal Tract and Tumors of the Annexed Organs

Gastrointestinal tumors, along with tumors of the annexed organs, are among the most diagnosed tumors worldwide. At the first position there is colon cancer, considered in the Global Cancer Statistic 2020 the fifth cancerous malignancy among the 36 common cancers, with new cases incidence of 6%, followed by stomach, liver, and rectum cancers. Pancreas cancer is less diagnosed, with 2.6% of new cases of incidence but 4.7% of mortality rate [67]. Surprisingly, based on the collected literature of the last two years and half, Eg5 appears to be a common altered marker in different gastrointestinal tumors.

Bioinformatic analysis of the TCGA data set revealed 29 upregulated kinesins in colon cancer, with strong overexpression of KIF11 in cancerous colon tissues compared to normal tissues. The higher expression of KIF11 is also related to the higher grades of tumor clinical stage T, M, and TNM, but not with stage N. A substantial reduction in *in vivo* and *in vitro* tumor growth is recorded after KIF11 knockdown, and, interestingly, silencing of KIF11 increased the sensitivity of colon cancer cells to oxaliplatin, a common anticancer drug used in colon cancer treatment; in fact, a reduction in the IC_50_ value of oxaliplatin and colony formation was recorded. This effect could be due to an aberrant activation of p53 pathway and a deactivation of GSK3*β* signaling, both effects triggered by KIF11 removal [31]. KIF11, together with KIF14, another kinesin involved in the correct cell division, could be responsible for the pathological genomic instability of colon cancer, and their levels reflect the clinical outcome. Both these kinesins could be used to stratify patients with better and worse overall survival: overall survival is negative when high KIF11 protein levels, high KIF11 protein together with low KIF14 protein levels, or low KIF11 and KIF14 mRNA levels, were found [32]. Eg5 is also a potential metastatic marker in T3 stage colon cancer, together with NEK9. The proteins of NEK family are cell cycle dependent proteins; since years it is well known that polo-like kinase 1, in collaboration with cyclin-dependent kinase 1, early phosphorylates NEK9 in mitosis, which in turn activates the NEK6/NEK7, members of the NEK family that phosphorylate Eg5 at S1033 in its tail domain, allowing its motor function and establishing a bipolar spindle [76, 77]. An overexpression of NEK9-Eg5 is recorded in patients with colon adenocarcinoma along with a distant metastasis association, thus representing a new possible biomarker to predict the metastatic potential in patients with T3 colon cancer [33].

The new prognostic model, based on a gene signature developed by Chu and co-workers, shows a 9-gene signature to predict the overall survival of patients with gastric cancer in Asian population: among 9 identified genes, KIF11 emerged [35] and these data could be extended to other populations. In fact, in a previous bioinformatic analysis, carried out by Ji et al., through the TCGA gastric adenocarcinoma dataset, 10 main genes of gastric cancer were highlighted, among which KIF11 is included [34], thus explaining why researchers are still focused on the research of the best Eg5 inhibitor to treat gastric cancer [36]. Moreover, an important role for Eg5 is also revealed in controlling *in vitro* nonmitotic processes directly related to the aggressiveness of gastric cancer, such as angiogenic and migratory events. Indeed, efficient pharmacological inhibition of Eg5 determined a substantial reduction of AGS gastric adenocarcinoma cells and a negative modulation of the angiogenic event, presumably through the involvement of PI3K-Akt-VEGF and Erk-VEGF pathways [37]. At the same time, this pharmacological inhibition acts on Eg5 mitotic functions, obtaining a valid reduction in AGS proliferation, with monoaster formation [78].

Hepatocellular carcinoma (HCC) is one of the cancer forms possessing the highest correlation with KIF11 overexpression. In the diagnosis and screening of HCC, serum alpha-fetoprotein is commonly used as molecular marker [79], however it is urgent to find new possible upregulated markers. 14 overexpressed hub genes were identified through an extensive and detailed bioinformatic algorithm and KIF11 is found among them. In this tumor model, upregulation of these genes is related to a worse overall survival time of patients. Interestingly, as previously reported for LUAD, there is a thin, but important correlation between KIF11 overexpression and tumor infiltrating immune cells: CD^4+^ memory activated T cells, macrophage M0, T cell follicular helper, regulatory T cells have a significant positive correlation with KIF11 overexpression [38]. The bioinformatic results were confirmed using 108 samples of surgical resection of HCC: Eg5 mRNA expression levels allowed to divide patients' samples into three tertile groups with high, medium and low Eg5 expression levels. The results showed that overall survival and disease-free survival of patients with low levels of Eg5 mRNA expression are better compared to those with medium and high levels of Eg5, allowing to assume that high Eg5 expression could be associated with a poor prognosis of HCC [39]. Hu et al. confirmed that KIF11 is negatively correlated with overall survival and disease-free survival of HCC patients, and its expression is positively correlated with tumor size: *in vitro* cell proliferation and tumor growth are significantly inhibited after KIF11 knockdown [40]. The molecular mechanism underlying Eg5 and HCC is unclear and further research is required to elucidate it. Zheng and co-workers hypothesized a regulation of Eg5 expression controlled by P21-activated kinases 6 (PAK6), a regulatory protein of cell migration, cell-cell adhesion, and apoptosis. When PAK6 is depleted in *in vitro* and *in vivo* models of HCC, overexpression of Eg5 is found, with a resulting formation of a multipolar spindle and cell cycle progression. In contrast, a knockdown of Eg5 completely inverted cell cycle progression and multipolar spindle formation together with PAK6 overexpression are highlighted. These results revealed that the outcome of patients with HCC could be influenced by the interaction between PAK6 and Eg5, promoting tumor progression [41]. As previously reported for LUAD, lnc-RNAs could play a decisive role in the correlation between Eg5 expression and HCC: one lncRNA, namely SNHG1, is correlated with *in vitro* expression of 6 upregulated hub genes connected with cell cycle progression, including KIF11. Knockdown of SNHG1 decreased the expression of these genes, along with the viability of two HCC cell lines, and determined a phase G1 cell cycle arrest acting as competitive endogenous RNA. This study emphasizes another possible pathway involved in KIF11 in HCC [43]. Lastly, Cao and coworkers found that KIF11 overexpression in HCC could also be related to epigenetic modifications, such as DNA methylation; in fact, among the genes exhibiting hypomethylation KIF11 is included, confirming once again the strong relationship between Eg5 expression and HCC and shedding light on another possible mechanism able to explain this correlation [42].

Based on these evidences, HCC is one of the main tumors with high expression of Eg5. A wide range of biomarkers are available for HCC diagnosis, from protein antigens (such as DKK-1, GP73, EMA, OPN, and others), to enzymes and isoenzymes (DCP, GGT, AFU, and PON1) [80]. Eg5 could be a new valid biomarker to be added in the panel of protein markers and to be analyzed by immunohistochemistry after tumor tissue biopsy: high rates of Eg5 expression could modify the therapeutic approach, with the possibility to add an Eg5 inhibitor to the standard therapy. In addition, future researches could detect possible association between Eg5 expression levels and tumor stage or identify Eg5 as a potential metastatic marker: transcriptome analysis could be a valid approach for this type of identification [81].

Pancreatic adenocarcinoma (PAC) is the second tumor of the gastrointestinal tract for which literature in the last 2 years demonstrated that a link between Eg5 expression and tumor onset exists. Bioinformatic analysis revealed differentially expressed genes, among which KIF11 is included, correlated with tumorigenesis and PAC development, and confirming these assumptions in *in vitro* models of pancreatic ductal adenocarcinoma cell lines. Overexpression of KIF11 is related to a worse overall survival of PAC [46]. Gu and colleagues highlighted a link between KIF11 and the mevalonate (MVA) metabolic pathway: recent findings revealed a causal association between cholesterol intake and PAC, meaning that the risks of PAC are closely dependent on cholesterol metabolism. This research group showed an interaction between Eg5 and SREBP2, the main regulator of MVA: it seems that high expression of KIF11 increased the expression of the SREBP2 protein and reduced its ubiquitin-mediated degradation. Promotion of cell growth, migration, stemming and colony formation, mediated by KIF11, depending on SREBP2, is highlighted: *in vivo* analysis revealed that PDA growth with high expression of KIF11 is controlled by targeting MVA biogenesis. Collectively, these findings suggest that KIF11 could activate MVA cross-talk to promote PAC [45]. As in colon cancer, also in PAC the expression of KIF11 and/or KIF14 could be identified as a discrimination marker between patients with better and worse overall survival, independently of other relevant clinical risk factors [44].

### 2.3. Tumors of the Male and Female Reproductive Systems

According to collected literature from 2021 and 2023, female reproductive system tumors having the highest correlation with Eg5 are breast cancer and ovarian cancer, while for male reproductive system tumors, a slight association was found between Eg5 and prostatic cancer.

Breast cancer (BC), with 11.7% incidence and 6.9% mortality in 2020, represents the first widespread cancer among women [67] and the need for a new target to treat this malignancy stimulates researchers to find new possible diagnostic and prognostic biomarkers.

**Figure 6:**
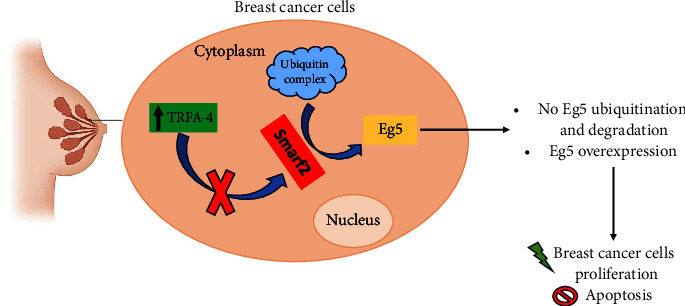
TRFA4/Smarf2/Eg5 is a possible axis involved in the onset of breast cancer. TRF4A overexpression blocks Smarf2-mediated Eg5 ubiquitination, with a reduction in Eg5 degradation and its consequent accumulation in cells, promoting proliferation of breast cancer cells and inhibition of apoptosis.

Through a bioinformatic analysis, differentially expressed genes correlated with cell division, cell proliferation, and BC signal transduction were identified. Among them, KIF11 emerged as associated with poor overall survival of patients [49, 52]. A possible signaling pathway that promotes BC proliferation through Eg5 activity is the TRFA4/Eg5 axis. TRFA4, acronym for Tumor Necrosis Factor Receptor Associated Factor 4, is an E3 ubiquitin ligase regulating the ubiquitination of different proteins. Both TRFA4 and Eg5 are overexpressed in BC and a positive correlation between them was found. Eg5 seems to interact with TRFA4 in the cytoplasm of BC cells, where the latter blocks the ubiquitination of Eg5 through an inhibition of Smarf2 (a promotor of Eg5 ubiquitination) activity. TRFA4 blocks the interaction of Smarf2/Eg5 inhibiting Eg5 ubiquitination catalyzed by Smurf2 and upregulating Eg5 levels, thus increasing and stabilizing Eg5 levels during mitosis (Figure 6). With this mechanism, BC proliferation is promoted and apoptosis inhibited [82].

KIF11 is also one of the 10 hub genes identified having a high and significant interaction with a BC marker, the thyroid hormone receptor interactor 13 (TRIP13), a protein acting in the spindle assembly checkpoint. The protein-protein interaction (PPI) network revealed that KIF11 could be a deregulator of TRIP13, representing a second possible therapeutic target [50].

Eg5 role in BC is also related to the molecular and histological classification of BC according to which three different types of BC can be recognized: BC positive for estrogen receptors (ER) and progesterone receptors (PR) (ER^+^, PR^+^), BC positive for ER, PR, and for human epidermal receptor 2 (HER2^+^), BC positive only for HER2 and triple negative BC (TNBC, negative for ER, PR, and HER2 receptors) [83]. KIF11 could be a target in TNBC, where its pharmacological inhibition revealed a slowdown in tumorigenesis and cancer progression in *in vivo* xenograft models. Interestingly, this pharmacological inhibition determined not only a downregulation of KIF11 expression, but also a downregulation of Aldehyde Dehydrogenase 1 family member A1 (ALDH1-A1): this is a marker of cancer stem cells (CSCs) and its high expression is correlated with poor overall survival [48]. A previous study demonstrated that endogenous administration of KIF11 promoted the self-renewal of BC cells through BC stem cells and that after silencing of KIF11 a decrease in CSC markers is highlighted, ALDH1 included [84]; as a consequence, it is possible to state that a positive correlation between KIF11 expression and cancer stem markers exists, thus representing a possible therapeutic target. Eg5 could also be a valid marker in ER^+^/PR^+^ BC: again, its pharmacological inhibition demonstrated a reduction in cell viability and proliferation probably correlated with the induction of apoptosis when Eg5 is completely inhibited: In parallel, a role for Eg5 in controlling events not related to mitotic activity, such as migration, invasion, and the occurrence of angiogenesis, was revealed. Indeed, after efficient pharmacological inhibition of Eg5 in an *in vitro* ER^+^/PR^+^ BC model, a slight inhibition of cell invasion was highlighted and a higher migration inhibition was reported, probably through a modulation of the MMP-9/NF-kB pathway. The transcription factor NF-kB, through a translocation into the nucleus, activates the transcription and protein secretion of the MMP-9 gene, which in turn is responsible for the degradation of the extracellular matrix, allowing cancer cells spreading. In ER^+^/PR^+^ BC model, represented by MCF7 cells, inhibition of Eg5 determined a downregulation of MMP-9 protein expression levels that reflects NF-kB protein levels, thus supposing a key role for Eg5/MMP-9/NF-kB axis in controlling cell migration. At the same time, a reduction in HIF-1^−^/VEGF protein expression levels was highlighted [51].

Considering the relevance of Eg5 in BC and the hypothetic mechanisms of action used by this protein to induce the onset or progression of tumor, it could be a new possible biomarker in addition to all the panel of markers already known: ER, PR, HER2, p5, Ki-67, BRCA1/BRCA2, PTEN, and others [85]. In particular, considering the variability of this tumor and the resistance to the current standard of care, the necessity to identify new biomarkers is still urgent [86]; Eg5 emerged as a new possible target, and it could be revolutionary for patients' treatment.

Using four microarray datasets, Zhao and colleagues investigated common differentially expressed genes in ovarian cancer. The KIF11 gene was identified among 20 genes highlighted through the PPI network and among the 6 genes whose overexpression is related to worse overall survival and progression-free survival in patients with ovarian cancer, together with a second kinesin called KIF23. The bioinformatic obtained results have been validated by an *in vitro* ovarian cancer model, represented by SKOV3 cell line: silencing of KIF11 results in a significant reduction of cell proliferation probably due to induction of apoptosis, as demonstrated by the increased activation of Caspase 3/7. Furthermore, as previously reported for other tumor models, KIF11 knockdown determines the effect not only on mitotic activity, but also on nonmitotic activity: siKIF11 reduced the invasion of SKOV3 cells and modified the expression levels of epithelial to mesenchymal transition genes, such as an upregulation of E-cadherin mRNA expression and a downregulation of N-cadherin and vimentin mRNA levels [54]. The bioinformatic results were also confirmed by Dong et al. [53], suggesting that further investigation is required to better clarify Eg5 role in this malignancy.

Regarding tumors of male reproductive system, prostatic cancer (PC) is the third for the number of new cases (7.3% incidence), after breast and lung cancer, in the Global Cancer Statistics 2020 [67]. Recent bioinformatic analysis of TCGA datasets, including RNA-Seq data from prostate adenocarcinoma, allowed the identification of KIF11 among genes having the highest significant correlation with PC. PC cell differentiation is determined by using the Gleason score, a number ranging from 2 to 10 identifying how many prostatic cells in tumor tissue are different from normal tissue, with the objective of establishing the aggressiveness of the tumor: values of 8 or higher correspond to a poorly differentiated tumor and are associated with a poor prognosis [87]. KIF11 gene expression was previously shown to be increased in PC samples having a Gleason score of 8 compared to samples with a Gleason score of 7 (moderately differentiated tumors) [88]. Pudova and colleagues found a strong negative correlation between KIF11 expression and tumor progression-free survival, thus confirming the fact that an Eg5 expression leads to worse PC prognosis for patients compared to those with lower levels of Eg5 [55]. Additionally, among the seven central genes, KIF11 could also represent a marker of bone PC metastasis: in fact, it was found a positive correlation between KIF11 and VEGF, both associated to poor metastasis-free survival (as a surrogate for overall survival). KIF11 and VEGF are together associated with higher T stage, with prostate specific antigen level, and with Gleason score [56].

### 2.4. Central Nervous System (CNS) Tumors

Eg5 is also upregulated in different CNS tumors, with a great extent for gliomas and the highest grade of glioma, glioblastoma (GBM), thus shedding light on the possibility of using this kinesin as a new possible target. Glioma data obtained with the TCGA database revealed 14–18 hub genes correlated with the worse survival prognosis and among them KIF11 is included [57, 62]. Upregulation of Eg5 is not only found in well-differentiated glioma cells, but also in more aggressive glioma stem cells (GSCs), as previously also revealed for other types of cancer. This subpopulation of cells with stemness and self-renewal characteristics, particularly aggressive in GBM, has the ability to escape from radiation and chemotherapy, constantly regenerating the tumor mass [89]. The expression of Eg5 is closely related to GSC markers [14]. Indeed, Liu and co-workers demonstrated that Eg5 promotes the expression of GSC markers, such as KLF4, OCT4, or NANOG, and the expression of the CD133 stemness marker, thus promoting the stemness associated with chemoresistance in TP53 mutant GBM. In addition, they found that Eg5 is associated with the expression of different cyclins, such as CDK1: the overexpression of Eg5 in the GBM model increased the expression of cyclins, modulating cell cycle progression [58]. Recently, Kenchappa and colleagues found a possible resistance mechanism developed in GBM to the administration of a well-known Eg5 inhibitor, ispinesib. They discovered a suppression of ispinesib apoptosis induction through double phosphorylation of STAT3. In GBM, STAT3 is a transcriptional regulator of the mesenchymal phenotype [90] which inhibits the induction of apoptosis and drives the self-renewal of cells, allowing chemoresistance [91, 92]. Two main phosphorylations are involved in the induction of resistance to ispinesib: the first phosphorylation interests the Y705 residue, performed by SCR proteins that inhibit STAT3-mediated transcription of genes related to antiapoptotic proteins. The second phosphorylation is on the S727 residue of STAT3, performed by the epidermal growth factor receptor (EGFR) which suppresses STAT3-mediated mitochondrial apoptosis induction. The resistance of GBM to Eg5 inhibitors could be due to the aforementioned phosphorylations: only one of the two phosphorylations is sufficient to induce a resistance mechanism to kinesin spindle protein inhibitors. A combined inhibition of SRC and EGFR with Eg5 inhibition could reverse this resistance process [59]. At the same time, targeting Eg5 could represent an approach to overcome GBM resistance to current standard of care: e.g., bevacizumab is an anti-VEGF antibody currently used in recurrent GBM, although resistance phenomena are responsible for a reduction of efficacy. Among the key regulators of resistance to bevacizumab, phosphofructokinase-1 muscle isoform (PFKM), emerged with Eg5 is an essential partner to trigger this molecular phenomenon: cytosolic PFKM interacts with Eg5, which, in turn, promotes GBM invasion [61]. Lastly, pharmacological inhibition of Eg5 in head and neck squamous cell carcinoma determined a blockage of cell proliferation by inducing a G2 cell cycle arrest and accumulation of cyclin B1 [60].

### 2.5. Others

Collected literature referring to 2021–2023 period reports other cancerous malignancies associated with Eg5. In bladder cancer, the bioinformatic analysis screened revealed 55 genes upregulated and 86 downregulated. KIF11 is one of the most significant biomarkers among all and it could be a promising new prognostic biomarker [63]. In cutaneous squamous cell carcinoma, immunohistochemical analyses highlighted a 70% of differences in Eg5 expression compared to normal skin, disclosing a high expression of this kinesin [64]. Immunohistochemical tissue analyses of 90 patients affected by clear cell renal cell carcinoma, followed for 7 years, revealed a high expression of KIF11 in the cytoplasmic fraction compared to the nuclear fraction, and this was positively correlated with tumor grade and mortality [65]. Lastly, Eg5 appears to have developed into a diagnostic and prognostic tool for thyroid carcinoma: patients with the highest KIF11 levels had the worst clinical pathological features (T stage and intraglandular dissemination). KIF11 inhibition suppressed thyroid cancer cell proliferation, triggering the apoptotic pathway. Furthermore, KIF11also appeared to help the development of thyroid cancer tumors in mice.

## 3. Eg5 in Noncancerous Diseases

As previously extensively reported, the mitotic and nonmitotic functions of Eg5 and their relative inhibition are usually associated with cancer. However, Eg5 could also play a role in other types of diseases. The main associations found in the literature are related to Eg5 expression and AD, microcephaly conditions (in particular, a specific condition characterized by microcephaly with or without chorioretinopathy, lymphedema, or intellectual disability, MCLID), and retinopathies (including familial exudative vitreous retinopathy, FEVR), even if additional pathological situations are reported in which Eg5 emerged. In this second part of the review, the authors will analyze the literature on less common topics.

### 3.1. Alzheimer's Disease

AD is a neurodegenerative disease for which specific causes have not yet been elucidated. There is a genetic form of AD, defined autosomal dominant AD (ADAD) or sporadic AD (SAD). Classic symptoms of AD start when the two main lesions in the CNS (the accumulation of beta-amyloid peptides (A*β*), with the formation of amyloid plaques [93] followed by the hyperphosphorylation of Tau protein, which determines the formation and accumulation of neurofibrillary tangles (NTF) [94]) affect neurons involved in memory, cognition, and neurogenesis. Lesions alter the normal structure and function of the CNS. Despite the extensive research focused on this topic, today pharmacological approaches are aimed at treating the symptoms' onset; however, improving early disease identification in the preclinical stage is a current challenge for researchers [95].

**Figure 7:**
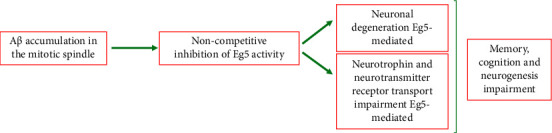
Effects of A*β* accumulation in AD patients and its inhibitory effect on Eg5 activity.

In contrast to what is reported about Eg5 and tumor onset and progression, in this type of disease the correct expression and function of Eg5 is essential to recover the AD deficit. Indeed, under AD conditions, cell cycle defects have been demonstrated [96]: almost 30% of aneuploid cells (in the CNS and peripheral tissues) are evident and premature centrosome division and chromosome mis-segregation are increased [97, 98]. In particular, an accumulation of A*β*, its precursor (the membrane-traversing amyloid precursor protein, APP), and enzymes normally cleaving the APP (the *β*-amyloid cleaving enzyme, BACE, and the presenilin (PS)-containing *γ*-secretase enzyme complex) were found in the mitotic spindle, especially at the poles of the spindle and kinetochores of neurons, and they appear particularly prone to degeneration. The mechanism by which the mitotic spindle is affected in AD, inducing neuronal degeneration, was discovered by Borysov et al. [99]. By using primary human somatic cells and cell-free Xenopus egg extract, they found that A*β* accumulation impairs mitotic spindle through an inhibition of some mitotic kinesins, in particular Eg5, KIF4A, and MCAK. The mechanism by which A*β* affects mitotic kinesin activity is different: MCAK is inhibited by a noncompetitive mechanism (direct inhibition of its activity), while inhibition of Eg5 and KIF4A occurs through a competitive mechanism, as A*β* interferes with the ability of both kinesins to interact with ATP and microtubules (Figure 7). The result is the formation of over 30% of aneuploid/hyperploid degeneration-prone neurons, interfering with neuronal function and plasticity.

Subsequently, it was also discovered that neuronal degeneration, primarily caused by Eg5 inhibition and subsequent mitotic spindle alteration, is further enhanced by inhibition of Eg5-mediated neurotrophin and neurotransmitter receptor transport to the cell surface (Figure 7). Indeed, as previously reported, Eg5 seems to participate in protein carriage through CARTS migration [11]. In AD, the amount of two essential receptors for the correct development and function of the CNS, the Nerve Growth Factor/Neurotrophin receptor (NGF/NTR) and the N-methyl-D-aspartate receptor (NMDA), appears to decrease on the surface of neurons after A*β* accumulation; this effect is comparable to that observed after chemical inhibition of Eg5 by monastrol. At the same time, neurons appear less susceptible to NGF with a reduction in neurite outgrowth, as well as Ca^2+^ entry into neurons regulated by the glutamate-dependent NMDA receptor [100]. In addition, A*β* accumulation is responsible for inhibition of long-term potentiation (LTP) NMDA-dependent, a key process for memory acquisition. When comparing the effect of A*β* and monastrol on LTP of the hippocampal slices, Ronald and co-workers found that monastrol can simulate A*β* effects on synaptic loss and LTP. In this way, they confirmed that A*β* causes acute and chronic synaptic dysfunction through inhibition of Eg5 [101], adding one more piece to the knowledge of the mechanism by which A*β* accumulation affects neuronal function in the disease.

A*β* accumulation is not the only cause of Eg5 inhibition and neuronal mitotic defects. In fact, as previously reported, the second main lesion recorded in AD patients is due to the formation of NTF by the hyperphosphorylated Tau protein. Tau is one of the main brain microtubule-associated proteins (MAP), predominantly in axons and neurons, with the main function of stabilizing the structure of MTs. This protein is highly expressed and phosphorylated not only in AD patients, but also in other neurodegenerative diseases, defined as tauopathies. A French research group from INSERM U1191 found that in AD Tau excess alters normal Eg5 function, with alteration in the mitotic process (monopolar spindle formation) and aneuploidy/apoptotic neuronal cell death [102]. Using a model of *Drosophila* developing wing disc epithelium (a columnar epithelium that during larval stages heads for several cell divisions to form future adult wing), they demonstrated that an excess of Tau protein induces mitotic arrest and monopolar spindle formation through a defect in the C-terminal Tau microtubule binding domain and an incorrect association of MTs during cell mitosis. However, they also found that these alterations in mitotic occurrence are due to specific mitotic kinesin, the Klp61F, the *Drosophila* homologue of human Eg5. Excess neuronal Tau affects the correct Klp61F location on MTs, inducing mitotic alterations. This result has been confirmed in an *in vitro* human model represented by epithelial HeLa cells, where the effect of Eg5 alteration, under Tau excess conditions, was also demonstrated [102].

**Figure 8:**
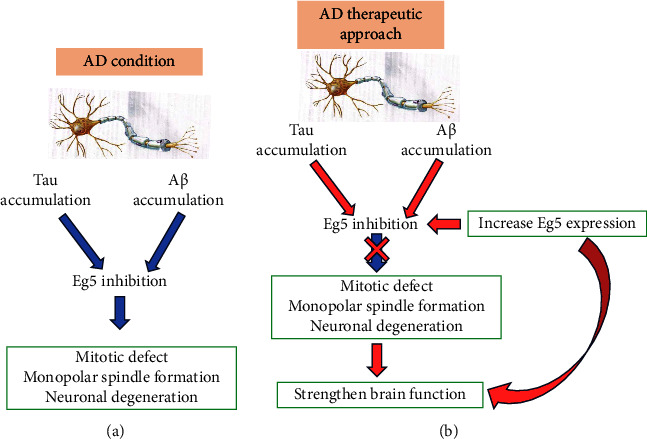
In AD condition (a) the accumulation of tau and A*β* blocks the normal activity of Eg5, leading to neuronal degeneration. A possible therapeutic approach (b) is to increase Eg5 expression to overcome its inhibition due to tau and/or A*β* accumulation.

Based on this knowledge, a recent study focused attention on the possibility of increasing Eg5 expression in AD to overcome its loss Tau/A*β*-mediated (Figure 8).

In rat primary neurons cell culture, KIF11 overexpression, obtained by transient transfection, strongly decreased the A*β*-mediated spine loss. In an *in vivo* model, represented by AD mouse model overexpressing KIF11, spatial learning and LPT deficits have been averted, improving learning and memory abilities, thanks to Eg5 upregulation. However, these positive effects after Eg5 overexpression do not decrease A*β* deposition. Therefore, Eg5 is a new possible target for drug development in AD: in particular, prevention of its complete inhibition by A*β* accumulation strengthens brain function, although the presence of deposits in AD brain still represents the main critical point of the disease [103].

### 3.2. Genetic Conditions Involving KIF11 Mutations

As previously specified, Eg5 is encoded by the KIF11 gene, located on the 10q23.33 chromosome. KIF11 alterations are responsible for some specific conditions, such as microcephaly and retinopathies. In particular, genetic mutations within KIF11 gene are responsible for two specific conditions: an autosomal dominant inherited disease characterized by microcephaly with or without chorioretinopathy, lymphedema, or intellectual disability (MCLID) and a familial exudative vitreous retinopathy (FEVR).

MCLID is a rare autosomal dominant condition described for the first time in 1992 [104]. In 2012, the association between this condition and KIF11 was reported for the first time: heterozygous KIF11 mutations were found in 15 of the 20 families affected by the disease, in particular two nonsense, two splice sites, four missense, and six indels causing frameshift mutations, were identified [105] and, subsequently, in 2014, five novel heterozygous mutations were detected [106].

From a phenotypic point of view, patients with MCLID present a different expression of symptoms: in a cohort of 37 individuals from 22 families, 86% were affected by microcephaly, 78% had an ocular abnormality, 46% had lymphoedema of the lower extremities, 73% had mild to moderate learning difficulties, 8% were affected by epilepsy and 8% had cardiac anomaly, demonstrating that there is a very high variability of phenotypic alterations [107]. The last update on this topic was reported in 2018 when, for the first time, a microdeletion was identified encompassing the entire KIF11 gene. It has been proven in two siblings, a boy and his sister, and in his father, all with true microcephaly, mild intellectual disability, and chorioretinopathy, at different levels [108].

The function of generated Eg5 protein in genetic MCLID KIF dependent gene is, of course, compromised: the microcephaly symptom could be due to the reduced activity of Eg5 in spindle formation, being this protein essential for organogenesis in general [3] and for neural development in particular [109]. However, the importance of KIF11 activity in the development and maintenance of retinal and lymphatic structures was highlighted by the fact that mutations in this gene were also detected in patients with chorioretinopathy and/or lymphedema, in addition to the microcephaly condition, supported by the finding that KIF11 is also localized in the inner segment and ciliary compartments of murine retina photoreceptor cells [110].

FEVR is the second genetic condition highly related to KIF11 mutations. It is a hereditary disease characterized by anomalous retinal vascular development with avascular peripheral retina. The consequence is a retinal vasculogenic disorder, with peripheral retinal nonperfusion, retinal folds, subretinal exudation, and detachment [111]. In 2014, 4 heterozygous mutations in the KIF11 gene were identified [109]. Other seven genes could be mutated and involved in FEVR pathogenesis (LRP5, FZD4, TSPAN12, NDP, CTNNA1, CTNNB1, and ZNF408); for this reason, Wang et al. recruited a cohort of patients with KIF11 mutations related to FEVR (35 patients from 25 families) to compare the KIF11-dependent FEVR phenotype to the FEVR patient phenotype with mutations in other possible genes (39 patients). Important evidence emerged from this study: 80% of patients with FEVR-related KIF11 mutation (20/25) showed variants of function loss, while 48% (12/25) of the variants were *de novo.* Phenotypically, chorioretinal dysplasia emerged in 44.2% of patients with the FEVR-related KIF11 mutation, while only 1.3% with other FEVR-related gene mutations. An increase and straightening of peripheral vessels (ISPV) was observed in 17.1% when KIF11 mutated and in 50% of FEVR with mutations in other genes. From this analysis, it was possible to conclude that chorioretinal dysplasia is the dominant phenotype in KIF11-associated retinopathy, while ISPV is common in FEVR with mutations in other genes [111–114].

Undoubtedly, KIF11 plays a role in retinal vascular development and its mutations alter the normal process. This could be associated with three important evidences: (1) Eg5 is correlated with angiogenesis, in cancer field, as previously described [13, 47, 66], but also in other noncancerous situations, such as retinopathy conditions; (2) Eg5 activity is essential for organogenesis. FEVR is one of the several pediatric vitreoretinopathies that can appear at birth or later; this means that the activity of this protein is essential for a correct retinal development; (3) KIF11 is also localized in the murine retina, as previously specified [110].

Although it is more common to find information on Eg5 and cancer when searching in common research motors (PubMed, Scopus), there are many available papers in which Eg5/KIF11 mutations are associated with microcephaly, and in particular, with MCLID and retinal pathological conditions, such as FEVR. In Table 2, a list of publications of the last 10 years is provided mainly focused on the genetic alterations of Eg5 and its pathological implications.

### 3.3. Other Noncancerous Diseases

Few articles associate other specific pathological conditions with Eg5 role; for each of them, only few scientific information is available, suggesting that further and deeper elucidations are required to officially associate Eg5 with other noncancerous diseases. One condition in which Eg5 appears to be involved is immune system alterations induced by human immunodeficiency virus type 1 (HIV-1). HIV is known to be responsible for acquired immune deficiency syndrome (AIDS) due to loss of CD^4+^ T-lymphocytes, with the consequent defects in immune system activity. Tat transactivation factor is one of the main and important proteins produced by HIV to regulate its proliferation, but also to induce apoptosis in CD^4+^ T-lymphocytes [137]. Liu and colleagues in 2014, by using two *in vitro* models of Jurkat cells and primary CD^4+^ T-lymphocytes from healthy donors, proposed a mechanistic explanation, Tat-Eg5 activity-mediated, by which HIV induces the disruption of T-lymphocytes [138]. They found that Tat, through its lysine 85 at the carboxyl terminus, acts as an allosteric inhibitor of Eg5 in CD^4+^ T-lymphocytes: by binding the allosteric site of Eg5, Tat inhibits protein ATPase activity through a block of ADP release and, therefore, determining a block of the entire ATP cycle, essential for Eg5 motor activity. As previously described, Eg5 activity inhibition provokes a block of the mitosis process, with formation of a monopolar spindle that culminates in apoptosis triggering. This happens also in CD^4+^ T-lymphocytes when Tat blocks Eg5 activity: the formation of the bipolar spindle is impaired with a block in cell cycle progression and apoptosis induction. A second study identified Eg5 as an essential driver of natural killer (NK) response to *Cryptococcus* infection in HIV patients. *Cryptococcus* is the main cause of fungal meningitis in HIV patients, and NK cells drive the cytolytic effect to remove it. Eg5, together with dynein, converge the NK cell granules, through MT structures, in the direction of *Cryptococcus* and controls the degranulation process, with the release of perforin, the main killing agent [139].

A novel bioinformatic analysis, aimed at identifying new possible biomarkers involved in pulmonary arterial hypertension, discovered KIF11 among the 10 hub genes involved in the disease, revealing that it is upregulated [140]. Additionally, in a study published in 2021, a correlation was also found between Eg5 and COVID-19: KIF11 is among the key genes altered in blood cells from patients infected, compared to normal blood cells [141].

## 4. Conclusions

This review summarizes the literature on Eg5 expression and functions in different pathological conditions. The main point emerging from this analysis is the dual effect of Eg5. In fact, this is the first review in which the role of Eg5 is elucidated taking into account not only its role in cancer, but also in other noncancerous diseases, opening new therapeutic frontiers.

On one hand, Eg5 overexpression or hyperactivity is responsible for different cancerous conditions, such as lung cancer and gastrointestinal tract carcinomas, followed by tumors of the reproductive system and of the central nervous system. This highlights the strong link existing between cancer onset/progression and Eg5 overexpression, classifying this kinesin as a new possible biomarker and a new therapeutic target. On the other hand, loss of function of Eg5, due to genetic alterations or structural/functional impairment, is responsible for different diseases, such as AD, microcephaly, or retinopathies, as well as AIDS, in which the association with Eg5 is less elucidated, thus identifying new frontiers to exploit.

## Figures and Tables

**Table 1:** The table resumes articles from 2021 to 2023 underlining a correlation between Eg5 expression and tumor onset and progression.

Tumor	Paper	Authors	Publication year
Lung cancer	KIF11 serves as an independent prognostic factor and therapeutic target for patients with lung adenocarcinoma	Li et al. [24]	2021
	Identification and integrate analysis of key biomarkers for diagnosis and prognosis of non-small-cell lung cancer based on bioinformatics analysis	Gong et al. [25]	2021
	Characterization of cell cycle-related competing endogenous RNAs using robust rank aggregation as prognostic biomarker in lung adenocarcinoma	Yang et al. [26]	2022
	LncRNA VPS9D1-AS1 promotes malignant progression of lung adenocarcinoma by targeting miRNA-30a-5p/KIF11 axis	Liu et al. [27]	2022
	Identification and verification of hub genes associated with the progression of non-small-cell lung cancer by integrated analysis	Mengyan et al. [28]	2022
	KIF11, a plus end-directed kinesin, as a key gene in benzo(a) pyrene-induced non-small-cell lung cancer	Ling et al. [29]	2022
	In silico analysis revealed the potential circRNA-miRNA-mRNA regulative network of non-small-cell lung cancer (NSCLC)	Balasundaram et al. [30]	2023

Colon cancer	KIF11 is upregulated in colorectal cancer and silencing of it impairs tumor growth and sensitizes colorectal cancer cells to oxaliplatin via p53/GSK3*β* signaling	Zhou et al. [31]	2021
	Prognostic impact and functional annotations of KIF11 and KIF14 expression in patients with colorectal cancer	Neska-Długosz et al. [32]	2021
	Overexpression of the NEK9-EG5 axis is a novel metastatic marker in pathologic stage T3 colon cancer	Kim et al. [33]	2023

Gastric cancer	Identification of the hub genes and prognostic indicators of gastric cancer and correlation of indicators with tumor-infiltrating immune cell levels	Ji et al. [34]	2021
	Bayesian hierarchical lasso cox model: A 9-gene prognostic signature for overall survival in gastric cancer in an Asian population	Chu et al. [35]	2022
	Synthesis of new 2-aminothiazolyl/benzothiazolyl-based 3,4-dihydropyrimidinones and evaluation of their effects on adenocarcinoma gastric cell migration	Sagha et al. [36]	2022
	Negative modulation of the angiogenic cascade induced by allosteric kinesin Eg5 inhibitors in a gastric adenocarcinoma *in vitro* model	Ricci et al. [37]	2022

Hepatocellular carcinoma	Identification of hub genes and their correlation with immune infiltration cells in hepatocellular carcinoma based on GEO and TCGA databases	Huang et al. [38]	2021
	Eg5 as a prognostic biomarker and potential therapeutic target for hepatocellular carcinoma	Shao et al. [39]	2021
	KIF11 promotes proliferation of hepatocellular carcinoma among patients with liver cancers	Hu et al. [40]	2021
	p21-activated kinase 6 controls mitosis and hepatocellular carcinoma progression by regulating Eg5	Zheng et al. [41]	2021
	Combined screening analysis of aberrantly methylated-differentially expressed genes and pathways in hepatocellular carcinoma	Cao et al. [42]	2022
	The SNHG1-Centered ceRNA network regulates cell cycle and is a potential prognostic biomarker for hepatocellular carcinoma	Zhou et al. [43]	2022

Pancreatic cancer	Prognostic significance of KIF11 and KIF14 expression in pancreatic adenocarcinoma	Klimaszewska-Wiśniewska et al. [44]	2021
	KIF11 manipulates SREBP2-dependent mevalonate cross talk to promote tumor progression in pancreatic ductal adenocarcinoma	Gu et al. [45]	2022
	Integrated bioinformatics analysis of potential biomarkers for pancreatic cancer	Shi et al. [46]	2022

Gallbladder cancer	KIF11 promotes cell proliferation via ERBB2/PI3K/AKT signaling pathway in gallbladder cancer	Wei et al. [47]	2021

Breast Cancer	Exploratory comparisons between different antimitotics in clinically-used drug combination in triple negative breast cancer	Guido et al. [48]	2021
	Screening and predicted value of potential biomarkers for breast cancer using bioinformatics analysis	Zeng et al. [49]	2021
	Evaluation of the TRIP13 level in breast cancer and insights into potential molecular pathways	Lan et al. [50]	2022
	Kinesin Eg5 selective inhibition by newly synthesized molecules as an alternative approach to counteract breast cancer progression: an *in vitro* study	Ricci et al. [51]	2022
	Identification of hub genes associated with breast cancer using integrated gene expression data with protein-protein interaction network	Elbashir et al. [52]	2023

Ovarian cancer	Integrative analysis of key candidate genes and signaling pathways in ovarian cancer by bioinformatics	Dong et al. [53]	2021
	Identification of the hub genes associated with the prognosis of ovarian cancer patients via integrated bioinformatics analysis and experimental validation	Zhao et al. [54]	2021

Prostatic cancer	Gene expression changes and associated pathways involved in the progression of prostate cancer advanced stages	Pudova et al. [55]	2021
	KIF11: A potential prognostic biomarker for predicting bone metastasis-free survival of prostate cancer	Wang et al. [56]	2022

Central nervous systems tumors	Glioma subtypes based on the activity changes of immunologic and hallmark gene sets in cancer	Chen [57]	2022
	Upregulation of KIF11 in TP53 mutant glioma promotes tumor stemness and drug resistance	Liu et al. [58]	2022
	Activation of STAT3 through combined SRC and EGFR signaling drives resistance to a mitotic kinesin inhibitor in glioblastoma	Kenchappa et al. [59]	2022
	The kinesin Eg5 inhibitor K858 exerts antiproliferative and proapoptotic effects and attenuates the invasive potential of head and neck squamous carcinoma cells	Nicolai et al. [60]	2022
	Nonmetabolic functions of phosphofructokinase-1 orchestrate tumor cellular invasion and genome maintenance under bevacizumab therapy	Lim et al. [61]	2022
	An *in silico* approach to the identification of diagnostic and prognostic markers in low-grade gliomas	Özbek et al. [62]	2023

Other tumors	Screening and identification of hub genes in bladder cancer by bioinformatics analysis and KIF11 is a potential prognostic biomarker	Mo et al. [63]	2021
	Screening and expression verification of key genes in cutaneous squamous cell carcinoma	Huang et al. [64]	2022
	Overexpression of kif11 is a poor prognostic factor in clear cell renal cell carcinoma	Kowalewski et al. [65]	2022
	KIF11 is a promising therapeutic target for thyroid cancer treatment	Han et al. [66]	2022

**Table 2:** List of publications of the last 10 years on the genetic correlation between KIF11 mutations and microcephaly/MCLID-retinopathies/FEVR.

Paper	Authors	Publication year
*KIF11 and microcephaly/MCLID*		
A novel KIF11 mutation in a Turkish patient with microcephaly, lymphedema, and chorioretinal dysplasia from a consanguineous family	Hazan et al. [115]	2012
Mutations in KIF11 cause autosomal-dominant microcephaly variably associated with congenital lymphedema and chorioretinopathy	Ostergaard et al. [105]	2012
Congenital microcephaly and chorioretinopathy due to de novo heterozygous KIF11 mutations: five novel mutations and review of the literature	Mirzaa et al. [106]	2014
Microcephaly with or without chorioretinopathy, lymphoedema, or mental retardation (MCLMR): review of phenotype associated with KIF11 mutations	Jones et al. [107]	2014
No evidence of locus heterogeneity in familial microcephaly with or without chorioretinopathy, lymphedema, or mental retardation syndrome	Schlögel et al. [116]	2015
Autosomal dominant microcephaly associated with congenital lymphedema and chorioretinopathy due to a novel mutation in KIF11	Mears et al. [117]	2015
Whole-exome sequencing is a powerful approach for establishing the etiological diagnosis in patients with intellectual disability and microcephaly	Rump et al. [118]	2016
Ocular manifestations of microcephaly with or without chorioretinopathy, lymphedema, or intellectual disability (MCLID) syndrome associated with mutations in KIF11	Balikova et al. [119]	2016
KIF11 microdeletion is associated with microcephaly, chorioretinopathy and intellectual disability	Malvezzi et al. [108]	2018
Detection and quantification of a KIF11 mosaicism in a subject presenting familial exudative vitreoretinopathy with microcephaly	Karjosukarso et al. [120]	2018
A novel mutation of KIF11 in a child with 22q11.2 deletion syndrome associated with MCLMR	Güneş et al. [121]	2019
Genotype phenotype correlation and variability in microcephaly associated with chorioretinopathy or familial exudative vitreoretinopathy	Shurygina et al. [122]	2020
First report of a de novo 10q23.31q23.33 microdeletion: Obesity, intellectual disability and microcephaly	Turkyilmaz et al. [123]	2021
KIF11 mutation with congenital microcephaly and chorioretinal lacunae	Shaikh et al. [124]	2022
A novel variant of the KIF11 gene, c.2922G > T, is associated with microcephaly by affecting RNA splicing. dev neurosci. 2022; 44 (2): 113–120	Guo et al. [125]	2022
Novel variant of KIF11 associated with MCLMR syndrome	Alahmadi et al. [126]	2023

*KIF11 and retinopathies/FEVR*		
Phenotypic overlap between familial exudative vitreoretinopathy and microcephaly, lymphedema, and chorioretinal dysplasia caused by KIF11 mutations	Robitaille et al. [112]	2014
Identification of novel KIF11 mutations in patients with familial exudative vitreoretinopathy and a phenotypic analysis	Li et al. [127]	2016
KIF11 mutations are a common cause of autosomal dominant familial exudative vitreoretinopathy	Hu et al. [128]	2016
Total retinal detachment caused by a KIF11 mutation	Riedl et al. [129]	2017
Novel insights into the phenotypical spectrum of KIF11-associated retinopathy, including a new form of retinal ciliopathy	Birtel et al. [110]	2017
Clinical and molecular characterization of familial exudative vitreoretinopathy associated with microcephaly	Hull et al. [130]	2019
Novel variants in familial exudative vitreoretinopathy patients with KIF11 mutations and the genotype-phenotype correlation	Chen et al. [131]	2020
A mouse model for kinesin family member 11- (Kif11-) associated familial exudative vitreoretinopathy	Wang et al. [132]	2020
Identification of potential molecular targets associated with proliferative diabetic retinopathy	Shao et al. [133]	2020
Retinal features of family members with familial exudative vitreoretinopathy caused by mutations in KIF11 gene	Kondo et al. [134]	2021
Severe exudative vitreoretinopathy as a common feature for CTNNB1, KIF11, and NDP variants plus sector degeneration for KIF11	Yang et al. [114]	2022
Update on the phenotypic and genotypic spectrum of KIF11-related retinopathy	Wang et al. [113]	2022
Phenotype-based genetic analysis reveals missing heritability of KIF11-related retinopathy: Clinical and genetic findings	Chang et al. [135]	2023
Mutations in the TSPAN12 and KIF11 genes in severe retinopathy of prematurity	Sun et al. [136]	2023

## Data Availability

No data were used for the research described in the article.
